# Accurate identification of fastidious Gram-negative rods: integration of both conventional phenotypic methods and 16S rRNA gene analysis

**DOI:** 10.1186/1471-2180-13-162

**Published:** 2013-07-16

**Authors:** Maria G de Melo Oliveira, Susanne Abels, Reinhard Zbinden, Guido V Bloemberg, Andrea Zbinden

**Affiliations:** 1 Institute of Medical Microbiology, University of Zurich, 8006 Zurich, Switzerland; 2 Present address, Policlínica Nascente, Prefeitura Municipal de Fortaleza, Fortalez-Ceará, Brasil; 3 Present address, Institute for Hygiene and Public Health, University Hospital, 53105 Bonn, Germany

**Keywords:** Fastidious Gram-negative rods, 16S rRNA gene, Conventional phenotypic methods

## Abstract

**Background:**

Accurate identification of fastidious Gram-negative rods (GNR) by conventional phenotypic characteristics is a challenge for diagnostic microbiology. The aim of this study was to evaluate the use of molecular methods, e.g., 16S rRNA gene sequence analysis for identification of fastidious GNR in the clinical microbiology laboratory.

**Results:**

A total of 158 clinical isolates covering 20 genera and 50 species isolated from 1993 to 2010 were analyzed by comparing biochemical and 16S rRNA gene sequence analysis based identification. 16S rRNA gene homology analysis identified 148/158 (94%) of the isolates to species level, 9/158 (5%) to genus and 1/158 (1%) to family level. Compared to 16S rRNA gene sequencing as reference method, phenotypic identification correctly identified 64/158 (40%) isolates to species level, mainly *Aggregatibacter aphrophilus*, *Cardiobacterium hominis*, *Eikenella corrodens, Pasteurella multocida*, and 21/158 (13%) isolates correctly to genus level, notably *Capnocytophaga* sp.; 73/158 (47%) of the isolates were not identified or misidentified.

**Conclusions:**

We herein propose an efficient strategy for accurate identification of fastidious GNR in the clinical microbiology laboratory by integrating both conventional phenotypic methods and 16S rRNA gene sequence analysis. We conclude that 16S rRNA gene sequencing is an effective means for identification of fastidious GNR, which are not readily identified by conventional phenotypic methods.

## Background

Accurate identification of fastidious Gram-negative rods (GNR) is a challenge for clinical microbiology laboratories. Fastidious GNR are slow-growing organisms, which generally require supplemented media or CO_2_ enriched atmosphere and fail to grow on enteric media such as MacConkey agar [1]. They are isolated infrequently and consist of different taxa including *Actinobacillus*, *Capnocytophaga*, *Cardiobacterium*, *Eikenella*, *Kingella*, *Moraxella*, *Neisseria*, and *Pasteurella*. Most of them are colonizers of the human oral cavity but they have been demonstrated to cause severe systemic infections like endocarditis, septicemia and abscesses, particularly in immunocompromised patients [1,2]. Accurate identification of fastidious GNR is of concern when isolated from normally sterile body sites regarding guidance of appropriate antimicrobial therapy and patient management [1].

Identification of fastidious GNR by conventional methods is difficult and time-consuming because phenotypic characteristics such as growth factor requirements, fermentation and assimilation of carbohydrates, morphology, and staining behaviour are subject to variation and dependent on individual interpretation and expertise [1,3]. Commercially available identification systems such as VITEK 2 NH (bioMérieux, Marcy L’Etoile, France) only partially allow for accurate identification of this group of microorganisms, e.g., *Eikenella corrodens, Kingella kingae* and *Cardiobacterium hominis*[4-6]. Most studies relied only on a subset of taxa of fastidious GNR or did not include clinical isolates under routine conditions [4-6]. The application of newer identification methods like matrix-assisted laser desorption ionization-time of flight mass spectrometry (MALDI-TOF MS) shows promising results regarding the identification of HACEK group members (*Haemophilus parainfluenzae*, *Aggregatibacter* spp., *Cardiobacterium* spp., *E. corrodens*, and *Kingella* spp.), however, only a small set of isolates and species were investigated [7-9]. Other potentially pathogenic fastidious GNR such as *Capnocytophaga* spp. or *Pasteurella* spp., which are known agents of wound infections and septicemia after animal bites [1] frequently are not included in comparative analyses. In addition, implementation of MALDI-TOF identification also depends on the number of correctly identified reference strains in the database.

16S rRNA gene sequence analysis is generally considered as the “gold standard” for bacterial identification [3,10,11]. We analysed a substantial data set of 158 clinical fastidious GNR isolates covering various difficult-to-identify taxa, which were collected during a 17-year period. We propose a feasible strategy for accurate identification of fastidious GNR in a routine diagnostic laboratory using both conventional phenotypic and molecular methods, e.g., 16S rRNA gene analysis.

## Methods

### Clinical isolates

The 158 isolates of fastidious GNR included in this study derived from clinical human specimens taken as part of standard patient care and were collected from 1993 to 2010 at the Institute of Medical Microbiology, University of Zurich, Switzerland. All isolates were identified both by conventional biochemical methods and 16S rRNA gene sequence analysis. The isolates were cultured on Columbia sheep blood or chocolate agar (Becton, Dickinson & Company, Franklin Lakes, NJ (BD)) and incubated at 37°C with 5% CO_2_ for 24 to 48 h. The isolates were stored at −80°C as pure cultures.

### Biochemical identification

The isolates were identified using in-house biochemical reactions as described for coryneform bacteria, for unusual Gram-negative aerobic bacteria and for facultative anaerobic bacteria [12,13]. In addition to the Gram stain, the following biochemical reactions were investigated: catalase, oxidase, nitrate reduction, urease, indole production, ornithine decarboxylase, hydrolysis of esculin; acid production from glucose, sucrose, maltose, mannitol and xylose was tested in semisolid cystine-trypticase agar medium (BD) supplemented with rabbit serum; tests for fermentative/nonfermentative carbohydrate metabolism were done on triple sugar iron agar. Identification by biochemical methods was scored as correct or incorrect taxonomic level compared to the 16S rRNA gene analysis as reference method. An incorrect assignment to species level was scored as incorrect species even if the genus was correct. If biochemical identification methods did not assign an isolate to at least genus level, the strain was scored as not identified.

### 16S rRNA gene sequence analysis

Sequencing of the partial 16S rRNA gene was performed as described previously [14]. In brief, a loopful of bacterial cells was used for extraction of DNA by lysozyme digestion and alkaline hydrolysis. Nucleic acids were purified using the QIAamp DNA blood kit (Qiagen AG, Basel, Switzerland). The 5’-part of the 16S rRNA gene (corresponding to *Escherichia coli* positions 10 to 806) was amplified using primers BAK11w [5´-AGTTTGATC(A/C)TGGCTCAG] and BAK2 [5´-GGACTAC(C/T/A)AGGGTATCTAAT]. Amplicons were purified and sequenced with forward primer BAK11w using an automatic DNA sequencer (ABI Prism 310 Genetic Analyzer; Applied Biosystems, Rotkreuz, Switzerland).

BLAST search of partial 16S rRNA gene sequences was performed by using Smartgene database (SmartGene™, Zug, Switzerland) on March 2013. The SmartGene database is updated with the newest 16S rRNA gene sequences from NCBI GenBank through an automated process every day. Non-validated taxa or non published sequences were not taken into consideration. The following criteria were used for 16S rRNA gene based identification [14-17]: (i) when the comparison of the sequence determined with a sequence in the database of a classified species yielded a similarity score of ≥ 99%, the isolate was assigned to that species; (ii) when the score was <99% and ≥ 95%, the isolate was assigned to the corresponding genus; (iii) when the score was < 95%, the isolate was assigned to a family. If the unknown isolate was assigned to a species and the second classified species in the scoring list showed less than 0.5% additional sequence divergence, the isolate was categorized as identified to the species level but with low demarcation. The sequence analysis was considered as the reference method but in cases with low demarcation results of supplemental conventional tests were taken into consideration for the final identification. Partial 16S rRNA gene sequences of all 158 clinical isolates were deposited in NCBI GenBank under GenBank accession numbers KC866143-KC866299 and GU797849, respectively.

### VITEK 2 NH card identification

A subset of 80 of the total of 158 isolates was tested by the colorimetric VITEK 2 NH card (bioMérieux) according to the instructions of the manufacturer. The colorimetric VITEK 2 NH card contains 30 tests and the corresponding database covers 26 taxa. Identification by VITEK 2 NH was compared to the 16S rRNA gene analysis as reference method.

## Results

One hundred fifty-eight clinically relevant human isolates of fastidious GNR (including rod forms of the genus *Neisseria*) were collected in our diagnostic laboratory during a 17-year period. Most of the 158 fastidious GNR isolates belonged to the following genera: *Neisseria* (n=35), *Pasteurella* (n=25), *Moraxella* (n=24), *Aggregatibacter* (n=20), *Capnocytophaga* (n=15), *Eikenella* (n=12), *Cardiobacterium* (n=6), *Actinobacillus* (n=3), *Oligella* (n=3)*,* and *Kingella* (n=2) (Table 1). 16S rRNA gene analysis identified 94% of the 158 isolates to species level and 5% to genus level; one isolate could only be assigned to family level (Tables 1 and 2). Thirteen isolates were assigned to species level with low demarcation to the next species but supplemental conventional tests revealed a final identification to species level (Table 1). Conventional methods assigned 60% of the isolates to species level and 15% to genus level (Tables 1 and 2). However, only 40% were correctly assigned to species level and 13% correct to genus level considering the 16S rRNA gene sequencing as reference method. 47% of the isolates were misidentified or not identified by conventional methods; nevertheless, 18 of the 31 isolates incorrectly assigned to species level were identified to the correct genus (Table 2).

**Table 1:** Identification of clinical isolates (n=158) by conventional methods compared to 16S rRNA gene sequence analysis

**Conventional phenotyic methods**		**16S rRNA gene sequence analysis**			**Final identification (supplemental conventional tests if required)**
**Identification (number of isolates)**	**Level of identification and correctness of result**	**Best reference species sequence**	**% difference to reference species sequence**	**GenBank accession numbers**	
*Actinobacillus ureae* (1)	S ^1^; SI ^2^	*Actinobacillus hominis Actinobacillus suis* (low demarcation)	0.0, 0.4	KC866152	*A. hominis* (acidification of mannitol: *A. hominis* (positive), *A. suis* (negative) [1])
*Aggregatibacter actinomycetemcomitans* (2)	S; SC	*Aggregatibacter actinomycetemcomitans*	0.0, 0.3	KC866227; KC866228	*A. actinomycetemcomitans*
*Aggregatibacter actinomycetemcomitans* (1)	S; SI	*Pasteurella bettyae*	0.0	KC866143	*P. bettyae*
*Aggregatibacter aphrophilus* (11)	S; SC	*Aggregatibacter aphrophilus*	0.0-0.8	KC866144; KC866145; KC866146; KC866147; KC866148; KC866149; KC866150; KC866229; KC866230; KC866231; KC866272	*A. aphrophilus*
*Aggregatibacter aphrophilus* (2)	S; SI	*Aggregatibacter aphrophilus*	3.8, 2.9	KC866151; KC866153	*Aggregatibacter* sp.
*Aggregatibacter aphrophilus* (1)	S; SI	*Neisseria sicca*	0.8	KC866154	*N. sicca* (nitrate reduction: positive (*N. mucosa*), negative (*N. sicca, N. subflava* bv. *flava*); sucrose acidification: positive (*N. sicca*, *N. mucosa*), negative (*N. subflava* bv. *flava*) [18])
		*Neisseria subflava* bv. *flava*	1.0		
		*Neisseria mucosa* (low demarcation)	1.1		
*Aggregatibacter* sp. (1)	G; GC	*Aggregatibacter aphrophilus*	2.3	KC866155	*Aggregatibacter* sp.
*Bergeyella zoohelcum* (1)	S; SI	*Myroides odoratimimus*	5.9	KC866156	*Flavobacteriaceae*
*Bergeyella zoohelcum* (1)	S; SI	*Neisseria zoodegmatis*	0.3	KC866157	*N. zoodegmatis*
*Capnocytophaga canimorsus* (2)	S; SC	*Capnocytophaga canimorsus*	0.5, 0.4	KC866158; KC866159	*C. canimorsus*
*Capnocytophaga ochracea* (1)	S; SI	*Capnocytophaga gingivalis*	0.6	KC866160	*C. gingivalis*
*Capnocytophaga ochracea* (1)	S; SI	*Capnocytophaga ochracea*	2.5	KC866161	*Capnocytophaga* sp.
*Capnocytophaga ochracea* (5)	S; SI	*Capnocytophaga sputigena*	0.0-0.3	KC866162; KC866163; KC866164; KC866273; KC866274	*C. sputigena*^3^
*Capnocytophaga ochracea* (1)	S; SI	*Dysgonomonas mossii*	0.6	KC866165	*D. mossii*
*Capnocytophaga ochracea* (1)	S; SI	*Leptotrichia trevisanii*	0.2	KC866166	*L. trevisanii*
*Capnocytophaga* sp. (2)	G; GC	*Capnocytophaga sputigena*	0.0, 0.6	KC866167; KC866232	*C. sputigena*
*Cardiobacterium hominis* (4)	S; SC	*Cardiobacterium hominis*	0.0-0.5	KC866168; KC866233; KC866275; KC866299	*C. hominis*
CDC Group IIe (1)	S; SI	*Chryseobacterium anthropi*	0.2	KC866169	*C. anthropi* (acidification of fructose and sucrose: positive (*C. haifense*), negative (*C. anthropi*) [19])
		*Chryseobacterium haifense* (low demarcation)	0.6		
*Comamonas* sp. (1)	G; GI	*Oligella urethralis*	0.0	KC866170	*O. urethralis*
*Dysgonomonas capnocytophagoides* (1)	S; SC	*Dysgonomonas capnocytophagoides*	0.2	KC866171	*D. capnocytophagoides*
*Eikenella corrodens* (10)	S; SC	*Eikenella corrodens*	0.0-0.8	KC866172; KC866173; KC866174; KC866175; KC866176; KC866177; KC866178; KC866234; KC866235; KC866236	*E. corrodens*
*Flavobacterium* sp. (1)	G; GC	*Flavobacterium lindanitolerans*	0.4	KC866179	*F. lindanitolerans*
Gram-negative rods (1)	N	*Actinobacillus hominis*	0.3	KC866238	*A. hominis*
Gram-negative rods (1)	N	*Actinobacillus hominis*	0.0	KC866237	*A. hominis* (esculin hydrolysis: positive (*A. suis*), variable (*A. hominis),* negative *(A. equuli*); mannitol acidification: positive (*A. equuli*, *A. hominis*), negative (*A. suis*) [1])
		*Actinobacillus suis*	0.0		
		*Actinobacillus equuli* (low demarcation)	0.5		
Gram-negative rods (1)	N	*Aggregatibacter actinomycetemcomitans*	0.2	KC866239	*A. actinomycetemcomitans*
Gram-negative rods (2)	N	*Aggregatibacter aphrophilus*	0.3, 0.8	KC866240; KC866241	*A. aphrophilus*
Gram-negative rods (1)	N	*Azospira oryzae*	0.0	KC866276	*A. oryzae*
Gram-negative rods (1)	N	*Brevundimonas terrae*	0.6	KC866180	*B. terrae*
Gram-negative rods (3)	N	*Capnocytophaga canimorsus*	0.0-0.2	KC866277; KC866278; KC866279	*C. canimorsus*
Gram-negative rods (1)	N	*Capnocytophaga sputigena*	0.0	KC866280	*C. sputigena*
Gram-negative rods (2)	N	*Cardiobacterium hominis*	0.5, 0.6	KC866281; KC866282	*C. hominis*
Gram-negative rods (1)	N	*Chryseobacterium haifense*	0.2	KC866181	*C. anthropi* (acidification of fructose and sucrose: positive (*C. haifense*), negative (*C. anthropi*) [19])
		*Chryseobacterium anthropi* (low demarcation)	0.5		
Gram-negative rods (1)	N	*Kingella denitrificans*	0.0	KC866182	*K. denitrificans*
Gram-negative rods (1)	N	*Moraxella atlantae*	0.2	KC866242	*M. atlantae*
Gram-negative rods (2)	N	*Moraxella lacunata*	0.0	KC866283; KC866284	*M. lacunata*
Gram-negative rods (1)	N	*Moraxella lincolnii*	0.3	KC866243	*M*. *lincolnii*
Gram-negative rods (3)	N	*Moraxella nonliquefaciens*	0.0-0.7	KC866285; KC866286; KC866287	*M. nonliquefaciens*
Gram-negative rods (2)	N	*Moraxella osloensis*	0.0, 0.2	KC866288; KC866289	*M. osloensis*
Gram-negative rods (1)	N	*Neisseria bacilliformis*	0.0	KC866244	*N. bacilliformis*
Gram-negative rods (1)	N	*Neisseria zoodegmatis*	2.0	KC866245	*Neisseria* sp.
Gram-negative rods (4)	N	*Neisseria elongata*	0.0-0.3	KC866246; KC866247; KC866290; KC866291	*N. elongata*
Gram-negative rods (1)	N	*Neisseria flavescens*	0.5	KC866248	*N. subflava* (acidification of glucose and maltose: positive (*N. subflava*), negative (*N. flavescens*) [18])
		*Neisseria subflava* (low demarcation)	0.7		
Gram-negative rods (2)	N	*Neisseria flavescens*	0.3	KC866249; KC866250	*N. subflava* (acidification of glucose and maltose: positive (*N. subflava*), negative (*N. flavescens*) [18])
		*Neisseria subflava* (low demarcation)	0.4		
Gram-negative rods (4)	N	*Neisseria weaveri*	0.0-0.3	KC866251; KC866252; KC866253; KC866254	*N. weaveri*
Gram-negative rods (1)	N	*Pasteurella bettyae*	0.0	KC866292	*P. bettyae*
Gram-negative rods (1)	N	*Pasteurella dagmatis*	0.4	KC866255	*P. stomatis* (urease reaction: positive (*P. dagmatis*), negative (*P. stomatis*); acidification of maltose: positive (*P. dagmatis*), negative (*P. stomatis*) [1])
		*Pasteurella stomatis* (low demarcation)	0.4		
*Kingella denitrificans* (1)	S; SC	*Kingella denitrificans*	0.6	KC866183	*K. denitrificans*
*Kingella denitrificans* (1)	S; SI	*Neisseria elongata*	0.0	KC866184	*N. elongata*
*Leptotrichia buccalis* (1)	S; SI	*Leptotrichia trevisanii*	0.3	KC866293	*L. trevisanii*
*Moraxella lacunata* (1)	S; SC	*Moraxella lacunata*	0.5	KC866185	*M. lacunata* (gelatinase reaction: positive (*M. lacunata*), negative (*M. nonliquefaciens*) [20])
		*Moraxella nonliquefaciens* (low demarcation)	0.7		
*Moraxella osloensis* (1)	S; SC	*Moraxella osloensis*	0.0	KC866186	*M. osloensis*
*Moraxella osloensis* (1)	S; SI	*Psychrobacter faecalis*	0.0	KC866187	*P. pulmonis* (acidification of glucose and xylose: positive (*P. faecalis*), negative (*P. pulmonis*) [20])
		*Psychrobacter pulmonis* (low demarcation)	0.2		
*Moraxella* sp. (1)	G; GC	*Moraxella canis*	0.2	KC866188	*M. canis*
*Neisseria* sp. (1)	G; GI	*Neisseria elongata*	0.3	KC866256	*N. elongata*
*Moraxella* sp. (4)	G; GC	*Moraxella nonliquefaciens*	0.0-0.3	KC866189; KC866190; KC866257; KC866258	*M. nonliquefaciens*
*Moraxella* sp. (8)	G; GC	*Moraxella osloensis*	0.0-0.2	KC866191; KC866192; KC866193; KC866194; KC866259; KC866260; KC866261; KC866294	*M. osloensis*
*Neisseria animaloris* (EF4a) (1)	S; SC	*Neisseria animaloris*	0.0	KC866195	*N. animaloris*
*Neisseria animaloris* (EF4a) (1)	S; SI	*Neisseria zoodegmatis*	0.0	GU797849	*N. zoodegmatis*
*Neisseria cinerea* (2)	S; SC	*Neisseria cinerea*	0.0	KC866196; KC866197	*N. cinerea* (acidification of glucose and maltose: positive (*N. meningitidis*), negative (*N. cinerea*) [18])
		*Neisseria meningitidis* (low demarcation)	0.3		
*Neisseria elongata* (1)	S; SI	*Aggregatibacter aphrophilus*	2.4	KC866198	*Aggregatibacter* sp.
*Neisseria elongata* (3)	S; SC	*Neisseria elongata*	0.0-0.3	KC866203; KC866204; KC866205	*N. elongata*
*Neisseria elongata* (2)	S; SI	*Neisseria bacilliformis*	0.1, 0.4	KC866201; KC866202	*N. bacilliformis*
*Neisseria elongata* (1)	S; SI	*Neisseria zoodegmatis*	0.6	KC866206	*N. zoodegmatis*
*Neisseria elongata* (2)	S; SI	*Eikenella corrodens*	0.0	KC866199; KC866200	*E. corrodens*
*Neisseria* sp. (1)	G; GC	*Neisseria shayeganii*	0.3	KC866207	*N. shayeganii*
*Neisseria* sp. (1)	G; GC	*Neisseria elongata*	0.2	KC866270	*N. elongata*
*Neisseria* sp. (1)	G; GC	*Neisseria oralis*	0.0	KC866208	*N. oralis*
*Neisseria weaveri* (1)	S; SC	*Neisseria weaveri*	0.0	KC866211	*N. weaveri*
*Neisseria weaveri* (1)	S; SC	*Neisseria shayeganii*	0.2	KC866210	*N. shayeganii*
*Neisseria weaveri* (1)	S; SI	*Azospira oryzae*	0.0	KC866209	*A. oryzae*
*Neisseria zoodegmatis* (EF4b) (3)	S; SC	*Neisseria zoodegmatis*	0.0-0.5	KC866212; KC866213; KC866295	*N. zoodegmatis*
*Oligella urethralis* (2)	S; SC	*Oligella urethralis*	0.0	KC866214; KC866215	*O. urethralis*
*Pasteurella aerogenes* (1)	S; SI	*Pasteurella aerogenes*	2.7	KC866226	*Pasteurella* sp.
*Pasteurella bettyae* (2)	S; SC	*Pasteurella bettyae*	0.0	KC866216; KC866262	*P. bettyae*
*Pasteurella canis* (1)	S; SC	*Pasteurella canis*	0.0	KC866217	*P. canis*
*Pasteurella canis* (1)	S; SI	*Pasteurella stomatis*	1.6	KC866218	*Pasteurella* sp.
*Pasteurella dagmatis* (1)	S; SC	*Pasteurella dagmatis*	0.2	KC866271	*P. dagmatis*
*Pasteurella multocida* (14)	S; SC	*Pasteurella multocida*	0.0-0.2	KC866219; KC866220; KC866221; KC866222; KC866223; KC866263; KC866264; KC866265; KC866266; KC866267; KC866268; KC866296; KC866297; KC866298	*P. multocida*
*Pasteurella pneumotropica* (1)	S; SI	Bisgaard Taxon 22	1.7	KC866224	*Pasteurella* sp.
*Pasteurella* sp. (1)	G; GI	*Necropsobacter rosorum*	0.0	KC866269	*N*. *rosorum*
*Roseomonas* sp. (1)	G; GC	*Roseomonas mucosa*	0.0	KC866225	*R. mucosa*

^1^Assignment to taxonomic level: S = species, G = genus, N = not identified.

^2^Correctness of assignment: SC = correct at species level, SI = incorrect at species level, GC = correct at genus level, GI = incorrect at genus level, N = not identified.

^3^ Difficult differentiation of species in question by conventional tests.

**Table 2:** Summary of identification of fastidious GNR isolates (n=158)

**Identification procedure**	**% correct identification at taxonomic level**		**% incorrect assignment at taxonomic level or no identification**		
	**Species**	**Genus**	**Species**	**Genus**	**No identification**
16S rRNA gene sequence analysis	94% (n=148)	5% (n=9)	-	-	1% (n=1)
Conventional phenotypic methods	40% (n=64)	13% (n=21)	20% (n=31)	2% (n=3)	25% (n=39)

Conventional methods mostly misidentified *Moraxella* spp. and *Neisseria* spp.; only 2 out of 24 *Moraxella* spp., 3 out of 10 *Neisseria elongata* and 1 out of 5 *Neisseria weaveri*, respectively, were correctly identified to species level. In contrast, results of phenotypic identification of *Aggregatibacter aphrophilus, Cardiobacterium hominis*, *E. corrodens*, *Pasteurella multocida* and *Capnocytophaga* sp. other than *Capnocytophaga canimorsus* were largely congruent with 16S rRNA gene sequence analysis (Table 3). These bacteria display biochemical key reactions that differentiate them from other fastidious GNR; e.g., a positive ornithine decarboxylase reaction and missing sugar acidification in the cystine-trypticase agar medium is typical for *E. corrodens*; a blood culture isolate with a positive indole reaction and a negative catalase is diagnostic for *C. hominis*; *P. multocida* has a typical pattern of acidification of sugars and a positive indole reaction and together with a history of cat bite the diagnosis is feasible [1]. *C. canimorsus* differs from *Capnocytophaga gingivalis*, *Capnocytophaga ochracea*, *Capnocytophaga sputigena* by the positive catalase and oxidase – together with the typical morphology of spindle-shaped cells in the Gram stain and the anamnestic history of a dog bite, the identification is possible with conventional methods; the other *Capnocytophaga* spp. with a negative catalase and oxidase are difficult to differentiate by conventional methods but identification to the genus level is feasible [21].

**Table 3:** Taxa with mostly reliable identification of fastidious GNR by conventional phenotypic methods

**Conventional phenotypic methods (number of isolates)**	**Final identification **^**1**^
*Aggregatibacter aphrophilus* (14)	*A. aphrophilus* (11)
	*Aggregatibacter* sp. (2)
	*Neisseria sicca* (1)
*Capnocytophaga canimorsus* (2)	*C. canimorsus* (2)
*Capnocytophaga* sp. (11)	*C. sputigena* (7)
	*C. gingivalis* (1)
	*Capnocytophaga* sp. (1)
	*Dysgonomonas mossii* (1)
	*Leptotrichia trevisanii* (1)
*Cardiobacterium hominis* (4)	*C. hominis* (4)
*Eikenella corrodens* (10)	*E. corrodens* (10)
*Pasteurella multocida* (14)	*P. multocida* (14)

^1^ Final identification was assigned using 16S rRNA gene identification as the reference method and if required with supplemental conventional tests.

The 80 out of 158 isolates analysed by the VITEK 2 NH card belonged to the following genera: *Neisseria* (n=21), *Moraxella* (n=13), *Eikenella* (n=12), *Aggregatibacter* (n=11), *Pasteurella* (n=9), *Capnocytophaga* (n=6), *Actinobacillus* (n=2), *Cardiobacterium* (n=2), *Kingella* (n=2), *Dysgonomonas* (n=1) and *Leptotrichia* (n=1) (Table 4). The VITEK 2 NH card identified 25 (31%) and 7 (9%) isolates to correct species and genus level, respectively; 4 isolates were assigned to incorrect genus and 21 isolates were not identified; 12 of the further 23 isolates incorrectly assigned to species level were identified to correct genus (Table 4). However, the VITEK 2 NH database includes taxa of only 43 of the 80 isolates studied. Regarding only taxa included in the VITEK 2 NH database, 25 (58%) and 7 (16%) out of 43 isolates were identified to correct species and genus level, respectively. The VITEK 2 NH card supports the identification of *A. aphrophilus, C. hominis, E. corrodens*, *Capnocytophaga* sp. and *Kingella* sp.

**Table 4:** Clinical isolates tested by the colorimetric VITEK 2 NH card (n=80)

**VITEK 2 NH card (number of isolates)**	**Level of identification and correctness of result**	**Final identification **^**1**^
*Actinobacillus ureae* (1)	S ^2^; SI ^3^	*A. hominis*
*Aggregatibacter aphrophilus* (5)	S; SC	*A. aphrophilus*^4^
*Aggregatibacter aphrophilus/Haemophilus parainfluenzae*^5^ (3)	G; GC	*A. aphrophilus*^4^
*Campylobacter fetus*/*coli* (2)	G; GI	*Moraxella osloensis*
*Capnocytophaga* sp. (4)	G; GC	*C. sputigena*^4^
*Capnocytophaga* sp. (1)	G; GI	*Dysgonomonas mossii*
*Capnocytophaga* sp. (1)	G; GI	*Leptotrichia trevisanii*
*Cardiobacterium hominis* (2)	S; SC	*C. hominis*^4^
*Eikenella corrodens* (11)	S; SC	*E. corrodens*^4^
*Eikenella corrodens* (1)	S; SI	*Neisseria elongata*^4^
*Haemophilus parainfluenzae* (1)	S; SI	*Actinobacillus hominis*
*Haemophilus parainfluenzae*^5^ (1)	S; SI	*Aggregatibacter aphrophilus*^4^
*Haemophilus parainfluenzae* (1)	S; SI	*Pasteurella multocida*^6^
*Kingella denitrificans* (2)	S; SC	*K. denitrificans*^4^
*Kingella denitrificans* (2)	S; SI	*Neisseria bacilliformis*
*Moraxella catarrhalis* (1)	S; SI	*M. nonliquefaciens*
*Moraxella catarrhalis* (2)	S; SI	*M. osloensis*
*Moraxella catarrhalis* (1)	S; SI	*Neisseria elongata*^4^
*Neisseria cinerea* (1)	S; SC	*N. cinerea*^4^
*Neisseria elongata* (1)	S; SI	*Capnocytophaga canimorsus*^4^
*Neisseria elongata* (1)	S; SI	*Capnocytophaga gingivalis*^4^
*Neisseria elongata* (1)	S; SI	*Eikenella corrodens*^4^
*Neisseria elongata* (3)	S; SC	*N. elongata*^4^
*Neisseria elongata* (4)	S; SI	*N. weaveri*
*Neisseria gonorrhoeae* (1)	S; SI	*Moraxella lacunata*
*Neisseria sicca* (1)	S; SC	*N. sicca*^4^
*Neisseria sicca* (2)	S; SI	*N. subflava*
*Neisseria elongata* (1)	S; SI	*N. zoodegmatis*
*Suttonella indologenes* (1)	S; SI	*Aggregatibacter actinomycetemcomitans*^4^
Not identified (1)	N	*Aggregatibacter aphrophilus*^4^
Not identified (1)	N	*Moraxella atlantae*
Not identified (1)	N	*Moraxella canis*
Not identified (3)	N	*Moraxella nonliquefaciens*
Not identified (2)	N	*Moraxella osloensis*
Not identified (1)	N	*Neisseria animaloris*
Not identified (3)	N	*Neisseria elongata*^4^
Not identified (1)	N	*Neisseria zoodegmatis*
Not identified (2)	N	*Pasteurella bettyae*
Not identified (5)	N	*Pasteurella multocida*^6^
Not identified (1)	N	*Pasteurella stomatis*

^1^ Final identification was assigned using 16S rRNA gene identification as the reference method and if required with supplemental conventional tests.

^2^ Assignment to taxonomic level: S = species, G = genus, N = not identified.

^3^ Correctness of assignment: SC = correct at species level, SI = incorrect at species level, GC = correct at genus level, GI = incorrect at genus level, N = not identified.

^4^ Taxon included in the VITEK 2 NH database; *Capnocytophaga* spp. is included as genus.

^5^ Accepted as correct genus as *Haemophilus aphrophilus* was renamed as *Aggregatibacter aphrophilus*[22].

^6^*Pasteurella multocida* is included in the database of the VITEK 2 ID GNB card (bioMérieux).

## Discussion

In this study, we analysed a large set of fastidious GNR clinical isolates covering diverse genera and species, which were obtained under routine conditions in a diagnostic microbiology laboratory. Molecular identification is vastly superior to conventional identification, both in number of isolates assigned to correct taxon level and in accuracy (Table 2). A minority (6%) of the 158 isolates included in the study could not be assigned to species level by 16S rRNA gene sequence analysis. In contrast, 47% of the 158 isolates were not identified or misidentified by conventional phenotypic methods (Table 2). However, the performance of supplemental phenotypic tests was helpful to support the molecular identification in cases with low demarcation of two or more species due to highly similar 16S rRNA gene sequences (Table 1).

Although the overall correct assignment to taxa by conventional phenotypic methods was rather poor, some species are easily assigned to correct species level by conventional identification procedures (Table 3). These are *A. aphrophilus, C. hominis*, *E. corrodens*, *P. multocida* and *Capnocytophaga* sp. other than *C. canimorsus*, which are characterised by typical biochemical key reactions that readily differentiate them from other fastidious GNR. In contrast, genera of *Moraxella* and *Neisseria* represent a challenge for the biochemical identification. Both genera often show similar biochemical reaction patterns, e.g., positive oxidase reaction or missing acid production from glucose, sucrose, maltose, mannitol, and xylose in semisolid cystine-trypticase agar medium; furthermore, the morphology in the Gram-stain does often not differentiate *Moraxella* and *Neisseria* species [13].

As alternative to conventional phenotypic methods, we analysed a subgroup of 80 isolates of fastidious GNR by the commercially available colorimetric VITEK 2 NH card (bioMérieux). Despite the limited database, this system supports the identification of fastidious GNR similar to that of conventional biochemical reactions by identifying 31% and 9% of the isolates to correct species and genus level, respectively.

Accurate identification of clinically relevant isolates of fastidious GNR is important for adequate interpretation and reporting as infectious agents and susceptibility testing [1]. However, in a routine diagnostic microbiology laboratory it is not feasible to subject all clinical isolates to molecular analyses for identification. Mahlen et al. proposed an efficient strategy by applying selective criteria such as discordant morphologic or biochemical results and knowledge of validity of phenotypic testing of isolates of Gram-negative bacilli [23]. Based on our data, we propose a cost-efficient algorithm, which is based on the knowledge of easy-to-identify organisms by conventional phenotypic methods and molecular analyses by the 16S rRNA gene for other difficult-to-differentiate species of this group. For identification of fastidious GNR conventional biochemical reactions and 16S rRNA gene sequence analysis can be implemented in a diagnostic laboratory as follows: (i) conventional biochemical identification of *A. aphrophilus, C. hominis, E. corrodens*, and *P. multocida* based on the typical reaction pattern is reliable; and (ii) any other result including *Capnocytophaga* sp. should be subjected to molecular methods by 16S rRNA gene analysis when accurate identification is of concern. By applying this approach to the 158 fastidious GNR analysed in our study, at least a third (32%) of the isolates would be readily identified by conventional phenotypic methods without laborious molecular analyses.

## Conclusions

In time of cost-effectiveness and rapid development of newer identification methods such as MALDI-TOF MS, an efficient strategy for difficult-to-identify bacteria is mandatory as alternative method. In this study we analysed a substantial set of various clinical isolates covering 20 genera and 50 species of fastidious GNR and evaluated the reliability of both conventional phenotypic methods and 16S rRNA gene analyses for accurate identification of such microorganisms. We propose an identification algorithm for fastidious GNR for a routine diagnostic laboratory as follows: (i) conventional biochemical identification of *A. aphrophilus, C. hominis, E. corrodens*, and *P. multocida* based on the typical reaction pattern is reliable; and (ii) any other result including *Capnocytophaga* sp. should be subjected to molecular methods by 16S rRNA gene analysis when accurate identification is of concern.

## Competing interests

The authors declare that they have no competing interests.

## Authors’ contributions

MMO contributed to the acquisition of laboratory data, analysis of biochemical data and drafting the manuscript. SA contributed to the overall study design and acquisition of molecular data. GVB contributed to the overall study design and critical revision of the draft. RZ contributed to the overall study design, analysis and interpretation of biochemical data and helped to draft the manuscript. AZ contributed to the acquisition of laboratory data, molecular analyses, evaluation of the sequence data and drafting the manuscript. All authors read and approved the final manuscript.
